# Vitamin B-6 and riboflavin, their metabolic interaction, and relationship with *MTHFR* genotype in adults aged 18–102 years

**DOI:** 10.1093/ajcn/nqac240

**Published:** 2022-10-20

**Authors:** Harry Jarrett, Helene McNulty, Catherine F Hughes, Kristina Pentieva, J J Strain, Adrian McCann, Liadhan McAnena, Conal Cunningham, Anne M Molloy, Albert Flynn, Sinead M Hopkins, Geraldine Horigan, Ciara O'Connor, Janette Walton, Breige A McNulty, Michael J Gibney, Yvonne Lamers, Mary Ward

**Affiliations:** Nutrition Innovation Centre for Food and Health (NICHE), School of Biomedical Sciences, Ulster University, Coleraine, United Kingdom; Nutrition Innovation Centre for Food and Health (NICHE), School of Biomedical Sciences, Ulster University, Coleraine, United Kingdom; Nutrition Innovation Centre for Food and Health (NICHE), School of Biomedical Sciences, Ulster University, Coleraine, United Kingdom; Nutrition Innovation Centre for Food and Health (NICHE), School of Biomedical Sciences, Ulster University, Coleraine, United Kingdom; Nutrition Innovation Centre for Food and Health (NICHE), School of Biomedical Sciences, Ulster University, Coleraine, United Kingdom; Nutrition Innovation Centre for Food and Health (NICHE), School of Biomedical Sciences, Ulster University, Coleraine, United Kingdom; Nutrition Innovation Centre for Food and Health (NICHE), School of Biomedical Sciences, Ulster University, Coleraine, United Kingdom; Department of Gerontology, St James’ Hospital, Dublin, Ireland; School of Medicine and School of Biochemistry and Immunology, Trinity College, Dublin, Ireland; School of Food and Nutritional Sciences, University College Cork, Cork, Ireland; Institute of Food and Health, University College Dublin, Dublin, Ireland; Nutrition Innovation Centre for Food and Health (NICHE), School of Biomedical Sciences, Ulster University, Coleraine, United Kingdom; Nutrition Innovation Centre for Food and Health (NICHE), School of Biomedical Sciences, Ulster University, Coleraine, United Kingdom; Department of Biological Sciences, Munster Technological University, Cork, Ireland; Institute of Food and Health, University College Dublin, Dublin, Ireland; Institute of Food and Health, University College Dublin, Dublin, Ireland; Food Nutrition and Health Program, Faculty of Land and Fo od Systems, University of British Columbia, Vancouver, Canada; Nutrition Innovation Centre for Food and Health (NICHE), School of Biomedical Sciences, Ulster University, Coleraine, United Kingdom

**Keywords:** vitamin B-6, riboflavin, pyridoxal 5′-phosphate, erythrocyte glutathione reductase activation coefficient, B-vitamin biomarkers, MTHFR, dietary intakes, Trinity-Ulster Department of Agriculture (TUDA)

## Abstract

**Background:**

The generation of the active form of vitamin B-6, pyridoxal 5′-phosphate (PLP), in tissues is dependent upon riboflavin as flavin mononucleotide, but whether this interaction is important for maintaining vitamin B-6 status is unclear.

**Objective:**

To investigate vitamin B-6 and riboflavin status, their metabolic interaction, and relationship with methylenetetrahydrofolate reductase (*MTHFR*) genotype in adulthood.

**Methods:**

Data from 5612 adults aged 18–102 y were drawn from the Irish National Adult Nutrition Survey (NANS; population-based sample) and the Trinity-Ulster Department of Agriculture (TUDA) and Genovit cohorts (volunteer samples). Plasma PLP and erythrocyte glutathione reductase activation coefficient (EGRac), as a functional indicator of riboflavin, were determined.

**Results:**

Older (≥65 y) compared with younger (<65 y) adults had significantly lower PLP concentrations (*P* < 0.001). A stepwise decrease in plasma PLP was observed across riboflavin categories, from optimal (EGRac ≤1.26), to suboptimal (EGRac: 1.27–1.39), to deficient (EGRac ≥1.40) status, an effect most pronounced in older adults (mean ± SEM: 76.4 ± 0.9 vs 65.0 ± 1.1 vs 55.4 ± 1.2 nmol/L; *P* < 0.001). In individuals with the variant *MTHFR* 677TT genotype combined with riboflavin deficiency, compared with non-TT (CC/CT) genotype participants with sufficient riboflavin, we observed PLP concentrations of 52.1 ± 2.9 compared with 76.8 ±0.7 nmol/L (*P* < 0.001). In participants with available dietary data (i.e., NANS cohort, *n* = 936), PLP was associated with vitamin B-6 intake (nonstandardized regression coefficient β: 2.49; 95% CI 1.75, 3.24; *P* < 0.001), supplement use (β: 81.72; 95% CI: 66.01, 97.43; *P* < 0.001), fortified food (β: 12.49; 95% CI: 2.08, 22.91; *P* = 0.019), and EGRac (β: –65.81; 95% CI: –99.08, –32.54; *P* < 0.001), along with BMI (β: –1.81; 95% CI: –3.31, –0.30; *P* = 0.019).

**Conclusions:**

These results are consistent with the known metabolic dependency of PLP on flavin mononucleotide (FMN) and suggest that riboflavin may be the limiting nutrient for maintaining vitamin B-6 status, particularly in individuals with the *MTHFR* 677TT genotype. Randomized trials are necessary to investigate the PLP response to riboflavin intervention within the dietary range. The TUDA study and the NANS are registered at www.ClinicalTrials.gov as NCT02664584 (27 January 2016) and NCT03374748 (15 December 2017), respectively.

Clinical Trial Registry details: Trinity-Ulster-Department of Agriculture (TUDA) study, ClinicalTrials.gov no. NCT02664584 (January 27th 2016); National Adult Nutrition Survey (NANS), ClinicalTrials.gov no. NCT03374748 (December 15th 2017).

See corresponding editorial on page 1472.

## Introduction

Vitamin B-6 and riboflavin play fundamental roles in numerous biologic processes, including one-carbon metabolism. Compared with the roles of folate and vitamin B-12 within this network, the metabolic and health effects of vitamin B-6 and riboflavin deficiency are much less well investigated in populations globally. Riboflavin status, in particular, is rarely assessed at a population level, but the limited available evidence suggests that deficiency may be more widespread than generally appreciated, including in high-income countries (1).

Riboflavin in its cofactor forms, FMN and FAD, is involved in the metabolism of energy, drugs, toxins, and other nutrients, including folate and vitamin B-6. The functional biomarker, erythrocyte glutathione reductase activation coefficient (EGRac), is used to assess riboflavin status, with higher values indicative of low or deficient status (2). The metabolically active form of vitamin B-6, pyridoxal 5′-phosphate (PLP), acts as a cofactor for enzymes required in multiple metabolic reactions (3, 4) and plasma PLP is the most commonly used biomarker for assessing vitamin B-6 status (5). The identification of modifiable factors that influence vitamin B-6 is important, considering that low plasma PLP is associated with increased risk of cardiovascular disease (6, 7), cancers (8–11), neurodegenerative diseases, cognitive impairment, anxiety, and depression (12–15), and appears to predict all-cause mortality (16).

Very few human studies have investigated the important metabolic interaction between riboflavin and vitamin B-6. Specifically, pyridoxine 5′-phosphate oxidase (PPO) requires riboflavin in the cofactor form of FMN for the deamination of pyridoxine 5′-phosphate and pyridoxamine 5′-phosphate to generate PLP (17). Animal studies indicate that PPO activity is sensitive to changes in riboflavin intake (18), with low PLP concentrations reported under conditions of riboflavin deficiency (19). In humans, consistent with its role in vitamin B-6 metabolism, our small intervention trial showed that riboflavin supplementation resulted in not only improved status of riboflavin (EGRac) but also increased plasma PLP in older adults with low status of either vitamin at baseline (20). Much more recently, we reported that riboflavin was significantly associated with PLP concentrations in healthy adults (21); the study was small, however, limiting the generalizability of our observations and extent to which the interrelationship of these nutrients could be investigated. Moreover, in other studies, we reported that riboflavin has a particular role in maintaining one-carbon metabolism, specifically in adults homozygous for the common C677T polymorphism in the*gene* encoding methylenetetrahydrofolate reductase (MTHFR) (22, 23), raising the possibility that riboflavin requirements may be increased in individuals with the variant TT genotype.

The primary aim of this study, therefore, was to investigate the association between biomarkers of vitamin B-6 and riboflavin status, and the interactive effect of *MTHFR* genotype, using data from large adult cohorts. In addition, the determinants of vitamin -B6 and riboflavin biomarkers were examined as a secondary outcome among participants with available dietary intake data.

## Methods

### Recruitment and study design

Data for this study were drawn from 3 cohorts: the National Adult Nutrition Survey (NANS) of Ireland, the Trinity-Ulster Department of Agriculture (TUDA) cohort study, and the Genovit case-control study (24). NANS is a nationally representative sample of Irish adults, with detailed dietary, nutritional status, and health and lifestyle data collected during the period of 2008–2010. Eligible participants were healthy adults, not pregnant or breastfeeding. Full sampling and methodological details for NANS have been reported previously (25). As described in detail elsewhere (26), the TUDA study comprises a cross-sectional cohort of 5186 older adults (≥60 y), with the primary aim of investigating nutritional factors in the development of chronic diseases of aging. Eligible participants were noninstitutionalized adults born on the island of Ireland. Participants were recruited during the period of 2008–2012 using standardized protocols, from general practitioner practices in the Northern and Western Trusts in Northern Ireland (United Kingdom) and from hospital outpatient clinics at the Department of Medicine for the Elderly at St. James's Hospital Dublin in the Republic of Ireland. The Genovit study included patients with cardiovascular disease recruited in 2003–2005 from the Cardiology Unit at Altnagelvin Area Hospital, Western Health and Social Care Trust, Northern Ireland, and apparently healthy, age- and sex-matched controls. Ethical approval for TUDA and Genovit were obtained from the Office for Research Ethics Committees Northern Ireland (reference number: TUDA, 08/NIR03/113, and Genovit, 08/NIR03/40) and/or from the Research Ethics Committee in St James's Hospital, and the Adelaide and Meath Hospital, Dublin. Ethical approval for NANS was obtained from University College Cork Clinical Research Ethics Committee of the Cork Teaching Hospitals and the Human Ethics Research Committee of University College Dublin [ECM 3 (p)], and all participants provided written informed consent at the time of recruitment.

### Dietary, lifestyle, and anthropometric data

For all study cohorts, health and lifestyle information was obtained in face-to-face interviews conducted by trained researchers using standardized protocols. Specifically, a comprehensive health and lifestyle questionnaire was administered to capture medical and demographic details, medications, and vitamin supplement usage. Weight, height, waist, and hip measurements were recorded; muscle mass was measured using the Tanita BC-420 (Tanita Ltd); and blood pressure was measured with a validated clinical automated blood pressure recording device (705 CP-II blood pressure monitor; Omron) in accordance with standard operating procedures.

In the NANS cohort detailed dietary intake data were collected as described elsewhere (25). Briefly, food and beverage intake data were recorded using a 4-consecutive-day semi-weighed food diary that included at least 1 weekend day. Participants were asked to record the type and amount of all food, beverages, and supplements consumed and, where applicable, record recipes, cooking method, and details of leftover food. Food intake data were analyzed using the food-composition database Weighed Intake Software Package (WISP) version 3.0 (Tinuviel Software) that uses data from McCance and Widdowson's “The Composition of Foods” sixth and fifth editions plus all 9 supplemental volumes to generate nutrient intake. Adjustments were made to the food-composition database to account for recipes, nutritional supplements, commonly consumed generic Irish foods, and new foods on the market.

### Blood sampling and laboratory analysis

Blood samples collected at the time of the appointment were analyzed for routine clinical measurements, including creatinine, triglycerides, HDL cholesterol, LDL cholesterol, high-sensitivity C-reactive protein (CRP), and hemoglobin in the participating local laboratories. B-vitamin status biomarkers across all cohorts were analyzed centrally in specialist research laboratories at Ulster University or Trinity College Dublin using standardized procedures.

PLP concentrations were measured as a biomarker of vitamin B-6 status as described in detail previously (27). Briefly, the method involved protein precipitation by trichloroacetic acid for release of PLP bound to protein, followed by conversion of PLP to 4-pyridoxic acid phosphate with cyanide in alkaline medium, acidification, separation by HPLC, and quantitation by a sensitive fluorescence detector. Despite a lack of consensus regarding cutoffs to define adequate vitamin B-6 status, plasma PLP concentrations <20.0 nmol/L and between 20.0 and 30.0 nmol/L are generally considered deficient and suboptimal, respectively (5). The latter cutoff was based on a controlled dietary intervention trial among healthy young adults, in which PLP values <30 nmol/L were associated with a wide range of metabolic effects, including perturbations of amino acid, lipid, and organic acid profiles in plasma (28). EGRac was assessed as a functional marker of riboflavin status (2) and represents the enzyme activity ratio for stimulated versus unstimulated glutathione reductase before and after in vitro activation with its prosthetic group, FAD. A higher EGRac ratio indicates lower riboflavin status. Although there are no universally accepted EGRac cutoffs to define optimal or low status (1), a coefficient of ≥1.40 was recently adopted to identify deficient riboflavin status as this is the cutoff generally used to denote deficiency in the few studies reporting this biomarker (29). Furthermore, in the absence of more robust evidence, a coefficient of ≤1.26 was adopted to define “optimal” riboflavin status based on the 95th percentile of the distribution of EGRac values measured in our previous study (30) after a 16-wk intervention with low-dose riboflavin (1.6 mg/d), an amount within the range of typical dietary intakes of riboflavin. In turn, an EGRac value between 1.27 and 1.39 was used to define suboptimal riboflavin status. RBC folate was measured by microbiological assay using *Lactobacillus casei* and serum total vitamin B-12 was also measured by microbiological assay using *Lactobacillus leichmanni*. Plasma homocysteine was measured by fluorescence polarization immunoassay.

Samples were analyzed blind. In the case of PLP, certified plasma controls at 2 different PLP concentrations (29.7 and 89.0 nmol/L; Chromsystems) were used. There is no commercially available quality control for RBC glutathione reductase (for riboflavin); therefore, stored in-house batches of pooled washed RBCs were used (2 aliquots for every run of 24 samples). Intra-assay and interassay CVs were ≤4.1% for plasma PLP and ≤4.5% for EGRac.

### Statistical analysis

**FIGURE 1:**
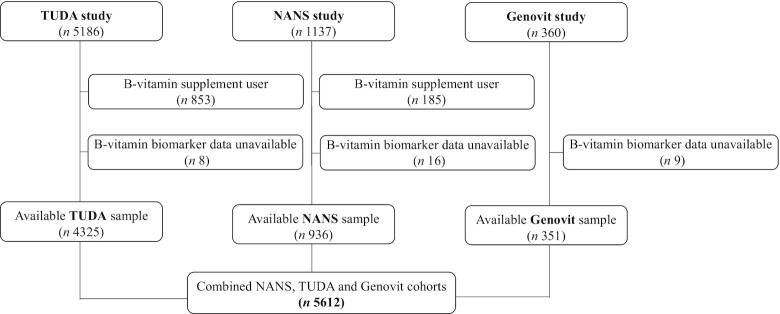
Study cohorts and flow chart of participants. NANS, National Adult and Nutrition Survey; TUDA, Trinity Department of Agriculture.

Statistical analysis was performed using the Statistical Package for the Social Sciences software (SPSS) (version 25.0; SPSS UK Ltd.). Before statistical analysis, tests for normality were performed and variables were log-transformed as appropriate. For the main analysis using the combined sample, B-vitamin supplement users and those without relevant B-vitamin biomarker data were excluded from the analysis (**Figure 1**). Analysis of the NANS cohort for intake-status relationships was conducted with and without the inclusion of supplement users.

Participant characteristics were compared between age groups (< 65 y vs. ≥65 y) by ANCOVA with Bonferroni post hoc tests, with adjustment for cohort, whereas categorical variables were assessed using chi-square analysis. The associations between age deciles and riboflavin and vitamin B-6 status were assessed using the Jonckheere-Terpstra test for trend. A 1-factor ANOVA with Scheffé post hoc tests was used to assess the impact of age on intake and biomarker status of vitamin B-6 and riboflavin in both males and females. Multiple linear regression analysis was performed to investigate the determinants of plasma PLP concentrations and EGRac. B-vitamin supplement use, dietary intakes of the respective vitamin, fortified food consumption, age, sex, BMI, alcohol intake, smoking, serum creatinine, serum CRP, hemoglobin, and muscle mass were considered as independent variables. For plasma PLP concentration, EGRac and energy intake were additionally included in the model, whereas for EGRac, milk consumption was also included. The relations among dietary and biomarker variables were examined in the 2 separate age groups (<65 y and ≥65 y), first with non–B-vitamin supplement users and then with B-vitamin supplement users included, by using Pearson partial correlation coefficients, controlling for age. ANCOVA with Bonferroni post hoc test was conducted in separate age and sex categories to investigate whether plasma PLP concentrations differed by riboflavin biomarker status groups as defined above (i.e., optimal, suboptimal, and deficient), after adjusting for age, BMI, and smoking status.

## Results

### Vitamin B-6 and riboflavin status and their association in healthy adults

Identification of the sample for analysis from the 3 observational cohorts is illustrated in Figure 1. The general characteristics of the study population are described in **Table 1**. With the exception of RBC folate, all variables were significantly different between younger (<65 y) and older (≥65 y) adults. For the B-vitamin biomarkers, younger adults had significantly higher plasma PLP and serum total vitamin B-12 concentrations along with lower plasma homocysteine concentrations. Older adults had better riboflavin status (i.e., lower EGRac values) (*P* ≤ 0.001). For vitamin B-6, 6% and 14% of younger and older adults were deficient (plasma PLP concentrations <30.0 nmol/L), respectively, whereas riboflavin deficiency (i.e., EGRac ≥1.40) was prevalent in both younger (39%) and older (29%) adults (data not shown).

**TABLE 1:** Characteristics of study participants^1^

	Participants aged <65 y (*n =* 1879)	Participants aged ≥65 y (*n =* 3733)	*P* ^2^
General characteristics			
Age, y	55.0 (42.0, 62.2)	75.5 (70.5, 81.6)	<0.001
Males, *n* (%)	956 (51)	1259 (34)	<0.001
BMI, kg/m^2^	27.5 (24.5, 31.0)	27.3 (24.2, 30.7)	<0.001
Waist circumference, cm	94.0 (84.0, 103.0)	95.0 (86.0, 104.0)	<0.001
Current smoker, *n* (%)	410 (22)	385 (10)	<0.001
Alcohol intake,^3^ units/wk	7.0 (2.0, 16.0)	3.0 (0.0, 8.0)	<0.001
Cardiovascular characteristics			
LDL cholesterol, mmol/L	2.63 (2.09, 3.26)	2.30 (1.78, 2.96)	<0.001
HDL cholesterol, mmol/L	1.36 (1.14, 1.67)	1.41 (1.15, 1.72)	0.007
Triglycerides, mmol/L	1.36 (0.92, 2.0)	1.36 (0.98, 1.91)	<0.001
hsCRP,^3^ mg/L	1.83 (0.89, 4.06)	2.58 (1.21, 5.95)	0.022
Creatinine,^3^ μmol/L	85.0 (73.0, 96.0)	82.0 (69.0, 99.0)	<0.001
Hypertensive,^4^*n* (%)	1025 (55)	3354 (90)	<0.001
Systolic BP, mmHg	131.5 (118.5, 145.5)	143.5 (130.0, 158.0)	<0.001
Diastolic BP, mmHg	80.5 (73.0, 87.5)	77.5 (70.0, 85.0)	<0.001
Antihypertensive drug use, *n* (%)	769 (41)	2915 (78)	<0.001
Statin drug use,^3^*n* (%)	437 (23)	1911 (51)	<0.001
B-vitamin biomarker status			
Vitamin B-6; plasma PLP, nmol/L	70.5 (49.1, 99.0)	58.1 (38.2, 85.5)	<0.001
Riboflavin; EGRac	1.35 (1.26, 1.47)	1.30 (1.21, 1.42)	<0.001
Vitamin B-12; serum total vitamin B-12,^3^ pmol/L	280 (206, 358)	256 (188, 340)	<0.001
Folate; RBC, nmol/L	844 (644, 1191)	908 (653, 1288)	0.523
Plasma homocysteine, µmol/L	11.6 (9.7, 14.0)	14.2 (11.6, 17.7)	<0.001

1 Values are medians (IQR) except where otherwise stated. *P* < 0.05 was considered significant. B-vitamin supplement users were excluded. BP, blood pressure; EGRac, erythrocyte glutathione reductase activation coefficient; hsCRP, high-sensitivity C-reactive protein; NANS, National Adult and Nutrition Survey; PLP, pyridoxal-5′-phosphate; TUDA, Trinity Department of Agriculture.

2 Differences between continuous variables analyzed by ANCOVA with Bonferroni post hoc tests, with adjustment for cohort. Differences between categorical variables were analyzed using chi-square analysis.

3 Data available for NANS and TUDA cohorts.

4 Hypertension defined as systolic BP ≥140 mmHg or diastolic BP ≥90 mmHg or taking BP medication.

**FIGURE 2:**
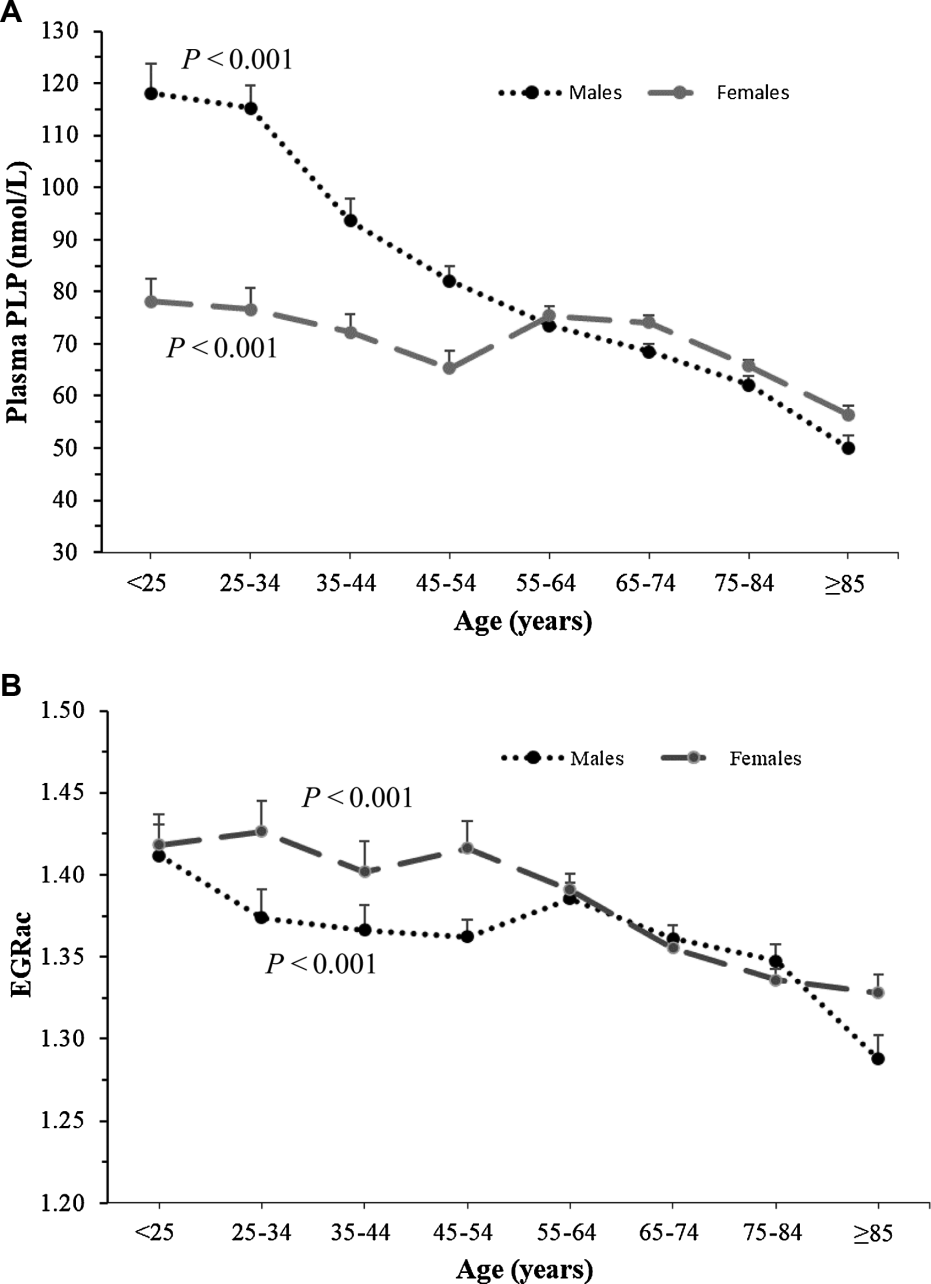
Plasma PLP concentrations (A) and EGRac (B) in unsupplemented adults (*n* = 5612; *n* = 2215 males, *n =* 3397 females). Data are expressed as means ± SEMs. *P* for trend calculated by Jonckheere-Terpstra test. EGRac, erythrocyte glutathione reductase activation coefficient; PLP, pyridoxal-5′-phosphate.

**FIGURE 3:**
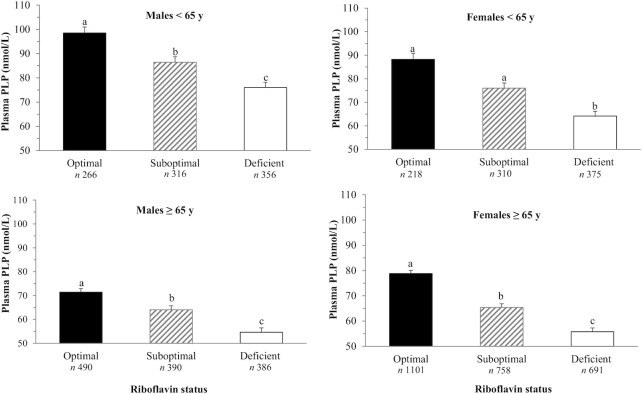
Plasma PLP concentrations stratified by riboflavin status in unsupplemented adults (*n =* 5549). Data are expressed as adjusted means ± SEMs. Differences were analyzed by ANCOVA adjusting for age, BMI, and cigarette smoking, with Bonferroni post hoc tests. Different superscript letters indicate significant differences, *P* < 0.001. Riboflavin status defined as optimal (EGRac ≤1.26) suboptimal (EGRac: 1.27–1.39), and deficient (EGRac ≥1.40). EGRac, erythrocyte glutathione reductase activation coefficient.

Plasma PLP concentrations were lower with increasing age for both males and females (*P*-trend < 0.001); however, this was most pronounced in males (**Figure 2**A), whereas a significant trend for better riboflavin status (indicated by lower EGRac values) was observed in older compared with younger adults in both males and females (*P*-trend < 0.001) (Figure 2B). In younger adults, males had better status of both vitamin B-6 and riboflavin compared with females, up to approximately 55 y of age. No significant interaction by sex was observed. There was a stepwise decrease in plasma PLP concentrations (nanomoles/liter) across riboflavin status categories from optimal (EGRac ≤1.26) to suboptimal (EGRac: 1.27–1.39) to deficient (EGRac ≤1.4) in both younger (93.8 ± 1.8, 81.8 ± 1.6, 69.6 ± 1.5; *P* < 0.001) and older (76.4 ± 0.9, 65.0 ± 1.1, 55.4 ± 1.2; *P* < 0.001) adults (data not shown). This stepwise decrease in plasma PLP concentrations across riboflavin status categories was observed in both males and females following adjustment for age, BMI, and smoking (**Figure 3**). The difference in plasma PLP concentrations across riboflavin status categories remained significant after adjustment for vitamin B-6 intake in the NANS cohort (*P* < 0.001) (data not shown).

### PLP, EGRac, and relationship with *MTHFR* genotype

**FIGURE 4:**
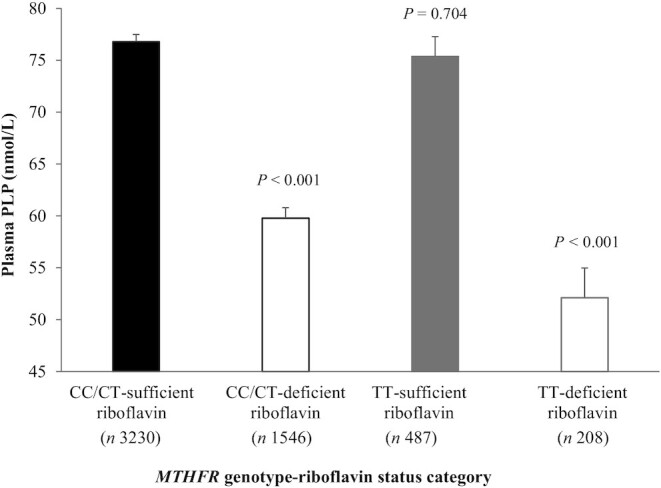
Plasma PLP concentrations stratified by *MTHFR* genotype in participants with sufficient vs. deficient riboflavin status. Riboflavin status defined as sufficient (EGRac <1.40) and deficient (EGRac ≥1.40). *P* values refer to comparisons of PLP concentrations of each *MTHFR* genotype–riboflavin category relative to CC/CT riboflavin-sufficient status category (reference category), analyzed by ANCOVA controlling for age. Comparison of PLP concentrations between TT riboflavin-deficient vs. CC/CT riboflavin-deficient individuals: *P* = 0.016. For this analysis, CC and CT genotype groups were combined as they are phenotypically similar, as we previously reported (41). EGRac, erythrocyte glutathione reductase activation coefficient; *MTHFR*, methylenetetrahydrofolate reductase; PLP, pyridoxal-5′-phosphate.

PLP concentrations were examined in four *MTHFR* genotype–riboflavin categories: non-TT sufficient, non-TT deficient, TT sufficient, and TT deficient (**Figure 4**). Compared with the CC/CT-sufficient riboflavin status as the reference category (76.8 ± 0.7), PLP concentrations (nanomoles/liter) were lowest (52.1 ± 2.87) in those with the variant *MTHFR* 677TT genotype combined with riboflavin deficiency (*P* < 0.001), but not significantly lower in the TT-sufficient riboflavin category (*P* = 0.704). **Supplemental Figure 1** shows plasma PLP concentrations stratified by *MTHFR* genotype in optimal compared with suboptimal riboflavin status.

### Dietary intake and status of vitamin B-6 and riboflavin—NANS cohort

Multiple linear regression analysis was performed to identify variables associated with the biomarkers of vitamin B-6 and riboflavin status in a subset of participants with available dietary intake data (i.e., NANS only and included supplement users) (**Table 2**). Plasma PLP concentrations were significantly associated with B-vitamin supplement use, dietary vitamin B-6 intake, fortified food consumption, BMI, and EGRac (Table 2). Age was also a significant determinant of vitamin B-6 status but only in non–supplement users (results not shown). For EGRac (Table 2), B-vitamin supplement use, dietary riboflavin intake, fortified food, milk consumption, age, smoking status, and hemoglobin were strongly associated with riboflavin status.

**TABLE 2:** Factors associated with vitamin B-6 and riboflavin status in Irish adults^1^

	Total cohort (*n =* 765)		
	β (95% CI)	β	*P*
Plasma PLP			
B-vitamin supplement use^2^	81.72 (66.01, 97.43)	0.35	<0.001
Dietary vitamin B-6 intake, mg/d	2.49 (1.75, 3.24)	0.22	<0.001
Fortified food consumption	12.49 (2.08, 22.91)	0.07	0.019
Energy, MJ/d	−1.18 (−3.40, 1.05)	−0.04	0.299
Age, y	−0.13 (−0.50, 0.23)	−0.03	0.469
Female sex	−0.99 (−24.70, 22.73)	−0.01	0.935
BMI, kg/m^2^	−1.81 (−3.31, −0.30)	−0.10	0.019
Alcohol, units/wk	0.02 (−0.05, 0.09)	0.02	0.621
Current smoker	−5.22 (−18.57, 8.13)	−0.02	0.443
Serum creatinine, µmol/L	0.06 (−0.38, 0.50)	0.01	0.786
Serum hsCRP, µmol/L	−1.68 (−3.55, 0.20)	−0.06	0.080
Hemoglobin, g/dL	0.98 (−3.76, 5.72)	0.02	0.686
Muscle mass, kg	0.83 (−0.28, 1.95)	0.11	0.142
EGRac	−65.81 (−99.08, −32.54)	−0.13	<0.001
Adjusted *R*²		0.30	
EGRac			
B-vitamin supplement use^2^	−0.07 (−0.11, −0.04)	−0.16	<0.001
Dietary riboflavin intake, mg/d	←0.01 (←0.01, <0.01)	−0.09	0.016
Fortified food consumption	−0.04 (−0.07, −0.02)	−0.12	0.001
Milk consumption	−0.04 (−0.07, −0.01)	−0.11	0.003
Age, y	←0.01 (←0.01, ←0.01)	−0.15	<0.001
Female sex	−0.01 (−0.06, 0.04)	−0.02	0.818
BMI, kg/m^2^	<0.01 (←0.01, <0.01)	0.01	0.795
Alcohol, units/week	<0.01 (←0.01, <0.01)	0.05	0.129
Current smoker	0.06 (0.03, 0.09)	0.14	<0.001
Serum creatinine, µmol/L	←0.01 (←0.01, <0.01)	−0.05	0.276
Serum hsCRP, µmol/L	<0.01 (←0.01, 0.01)	0.02	0.539
Hemoglobin, g/dl	−0.02 (−0.03, −0.01)	−0.14	0.001
Muscle mass, kg	<0.01 (←0.01, <0.01)	0.01	0.875
Adjusted *R*²		0.14	

1 Values are nonstandardized regression coefficients (β), corresponding 95% CIs, and standardized coefficients (β). Multiple linear regression analyses were performed using plasma PLP concentration and EGRac as dependent variables, respectively, using data from the National Adult Nutrition Survey (NANS) of Irish adults. In the plasma PLP model, B-vitamin supplement use (nonconsumers of B-vitamin supplements as reference category), dietary vitamin B-6 intake (mg/d), fortified food consumer (nonconsumers and lowest tertile of vitamin B-6 intake from fortified foods as reference category), age (y), sex (male as reference category), BMI (kg/m²), alcohol intake (units/wk), smoking (nonsmoker as reference category), serum creatinine (µmol/L), hsCRP (µmol/L), hemoglobin (g/dL, muscle mass (kg), and EGRac were included in the model as independent variables. In the riboflavin model, B-vitamin supplement use (nonconsumers of B-vitamin supplements as reference category), dietary riboflavin intake (mg/d), fortified food consumer (nonconsumers and lowest tertile of riboflavin intake from fortified foods as reference category), milk consumption (lowest quartile of milk intake as reference category) age (y), sex (male as reference category), BMI (kg/m²), alcohol intake (units/wk), smoking (nonsmoker as reference category), serum creatinine (µmol/L), hsCRP (µmol/L), hemoglobin (g/dL), and muscle mass (kg) were included in the model as independent variables. Higher EGRac values are indicative of lower status. EGRac, erythrocyte glutathione reductase activation coefficient; hsCRP, high-sensitivity C-reactive protein; PLP, pyridoxal 5´-phosphate.

2 Supplement users identified as those consuming supplemental B-vitamins (in tablet form) during food diary recording.

Intake and biomarker status of each B-vitamin were then compared across 3 age categories—18–50 y, 51–64 y, and ≥65 y—in males and females. Consistent with the data presented in Figure 2, males aged ≥65 y had significantly lower plasma PLP concentrations compared with males in the youngest age group (18–50 y) (*P* < 0.001), whereas there were no significant differences across age groups for females (*P* = 0.377). There were no differences among dietary vitamin B-6 intakes across the 3 age categories for either males (*P* = 0.112) or females (*P* = 0.430). Riboflavin status was better in older females aged ≥65 y compared with females in the youngest age group (18–50 y) (*P* = 0.003), whereas there were no significant differences in riboflavin status across the age groups for males (*P* = 0.105). Corresponding dietary riboflavin intakes were significantly higher for males in the youngest age group compared with males in the 2 older age groups (51–64 y and ≥65 y) (*P* < 0.001). There were no significant differences in dietary riboflavin intakes for females (*P* = 0.393) (**Table 3**).

**TABLE 3:** Dietary intakes and biomarkers of vitamin B-6 and riboflavin in younger and older adults^1^

	Age category			
	18–50 y (*n* = 628)	51–64 y (*n* = 184)	≥65 y (*n* = 124)	*P* ^2^
General characteristics				
Male, %	52	54	46	0.363
Age, y	34 (10)^a^	57 (4)^b^	72 (5)^c^	<0.001
BMI, kg/m^2^	26.3 (4.4)^a^	29.4 (5.1)^b^	27.7 (4.0)^c^	<0.001
Energy intake, MJ/d				
Males	10.5 (2.7)^a^	9.4 (2.5)^b^	8.1 (2.5)^c^	<0.001
Females	7.4 (2.2)^a^	7.1 (1.7)^a,b^	6.5 (1.5)^b^	0.024
Vitamin B-6				
Intake, mg/d				
Males	3.2 (1.1)	3.0 (1.1)	2.9 (1.4)	0.112
Females	2.1 (0.8)	2.2 (0.8)	2.3 (0.9)	0.430
Biomarker status (plasma PLP), nmol/L				
Males	105.8 (45.3)^a^	95.5 (44.2)^a^	74.3 (36.0)^b^	<0.001
Females	74.6 (35.3)	78.6 (43.1)	72.1 (44.4)	0.377
Riboflavin				
Intake, mg/d				
Males	2.3 (1.0)^a^	2.0 (0.8)^b^	1.8 (0.7)^b^	<0.001
Females	1.6 (0.6)	1.6 (0.5)	1.6 (0.7)	0.393
Biomarker status (EGRac)				
Males	1.38 (0.17)	1.35 (0.17)	1.34 (0.15)	0.105
Females	1.41 (0.17)^a^	1.38 (0.16)^a, b^	1.34 (0.16)^b^	0.003

1 Values are means (SDs) unless otherwise stated. Data available for NANS cohort *n =* 936, whereby corresponding biomarker and dietary data were available for unsupplemented adults. Values in a row without a common superscript letter are significantly different, *P* < 0.05 (Scheffé post hoc test). EGRac, erythrocyte glutathione reductase activation coefficient; PLP, pyridoxal 5´-phosphate.

2 General characteristics were compared between groups by using chi-square analysis and 1-factor ANOVA (Scheffé post hoc tests) for categorical and continuous variables, respectively. Differences in riboflavin and vitamin B-6 intake and biomarker status were analyzed by 1-factor ANOVA with Scheffé post hoc tests.

**FIGURE 5:**
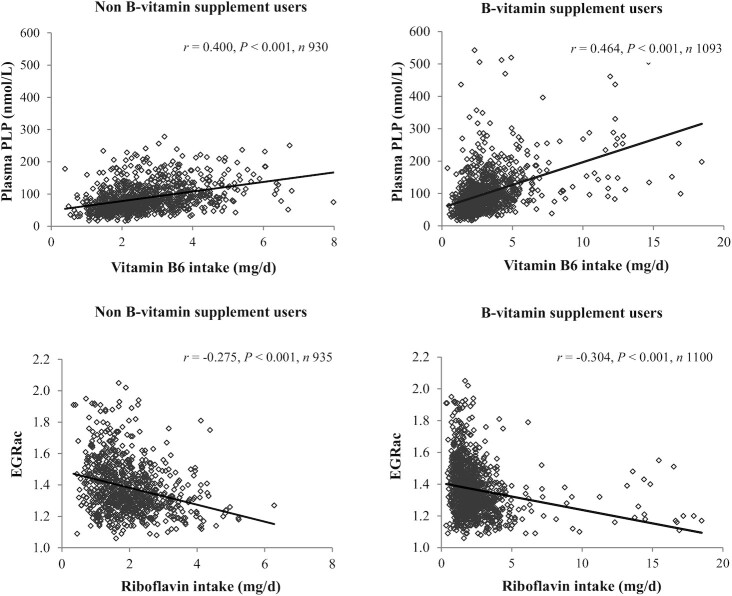
Relation of dietary intakes of vitamin B-6 (upper plots) and riboflavin (lower plots) with their corresponding biomarkers in Irish adults. Correlations were carried out on log-transformed data and calculated by using Pearson's partial correlation coefficients (*r*), adjusting for age. EGRac, erythrocyte glutathione reductase activation coefficient; PLP, pyridoxal-5′-phosphate.

In the NANS participants who did not use B-vitamin supplements the relationship between reported dietary intake of each B-vitamin and the corresponding status was examined. Strong correlations between dietary intake and status of vitamin B-6 (PLP; *r* = 0.400, *P* < 0.001) and riboflavin (EGRac; *r* = –0.275, *P* < 0.001) were observed. In the total NANS cohort, including B-vitamin supplement users, a stronger relationship between dietary intake and status for both vitamin B-6 (PLP; *r* = 0.464, *P* < 0.001) and riboflavin (EGRac; *r* = –0.304, *P* < 0.001) was demonstrated (**Figure 5**). Following inclusion of B-vitamin supplement users, the association between vitamin B-6 intake and plasma PLP (*r* = 0.392, *P* < 0.001) was greater when stratifying the cohort into younger adults (*r* = 0.451, *P* < 0.001) and older adults (*r* = 0.435, *P* < 0.001 vs. *r* = 0.536, *P* < 0.001) (results not shown). Similarly, the association between riboflavin intake and EGRac strengthened with the inclusion of B-vitamin supplement users in younger (*r* = –0.292, *P* < 0.001 vs. *r* = –0.303, *P* < 0.001) and older adults (*r* = –0.182, *P* < 0.044 vs. *r* = –0.329, *P* < 0.001) (results not shown).

## Discussion

In a sample of >5000 adults aged 18–102 y drawn from 3 observational cohorts, we show that plasma PLP is strongly associated with riboflavin status throughout adulthood, consistent with the known metabolic interaction between these 2 nutrients. In this large sample, PLP concentrations were found to decrease in a stepwise manner from optimal to suboptimal to deficient riboflavin status. Furthermore, the *MTHFR* C677T polymorphism interacted with riboflavin to influence PLP, in that the lowest PLP concentrations were found in individuals with riboflavin deficiency combined with the variant TT genotype.

The current results provide evidence of the metabolic dependency of PLP on FMN and are broadly consistent with the limited previous human studies that exist (20, 21, 31, 32). We found that plasma PLP was 24-nmol/L lower in participants categorized as having deficient compared with optimal riboflavin status, an observation that is entirely consistent with a trial we conducted some years ago, which showed an increase in plasma PLP by 24 nmol/L in response to riboflavin supplementation of older adults with baseline concentrations of PLP indicative of vitamin B-6 deficiency (20). Likewise, in a convenience sample of healthy adults, we recently observed a strong association of EGRac with PLP concentrations (21). The human evidence is in line with early animal studies demonstrating low PLP concentrations in rats fed a riboflavin-deficient diet (19) and responses in PPO activity to changes in riboflavin intake (18). The current finding of low PLP concentrations among those with deficient riboflavin status, independent of dietary vitamin B-6 intake, adds considerably to our earlier observational (21) and trial (20) findings. Furthermore, although previous studies have reported significant associations between vitamin B-6 intake and plasma PLP (33–35), here we showed that vitamin B-6 status declines with age across adulthood, particularly in males. Consistent with UK population–based data from the National Diet and Nutrition Survey (NDNS) (36), mean PLP concentrations were >30 nmol/L higher in younger compared with older males (36). Similarly, low plasma PLP has been reported in nonsupplemented older US males in the NHANES (*n* = 4463) (33, 37). The explanation for lower vitamin B-6 status in older males is unclear, given that dietary intakes were very similar to those in younger males, but could be related to increased vitamin -B6 catabolism due to low-grade chronic inflammatory processes associated with aging (38) or decreased protein binding capacity of plasma, leading to increases in free PLP (5). The decline in PLP concentrations with age was less pronounced in females, consistent with previous reports of sex-specific differences in plasma PLP trajectories with age (33, 37).

Consistent with the known metabolic dependency of PLP on FMN, our results highlight the importance of riboflavin status and suggest that it may be the limiting nutrient for maintaining adequate vitamin B-6 status, particularly among older people in whom PLP concentrations are lowest, as shown here and elsewhere (33, 36, 37, 39). We examined determinants of PLP using multiple linear regression in a subset of participants with available dietary data (i.e., NANS cohort) and showed that the strong association of EGRac with PLP was evident after adjustment for other important determinants—namely, dietary vitamin B-6 intake, supplement use, fortified food, and BMI. Of note, we found that PLP concentrations were lowest in those with riboflavin deficiency combined with the variant *MTHFR* 677TT genotype. This suggests that individuals homozygous for this common polymorphism may be most at risk of the functional consequences of riboflavin deficiency. Apart from the functional impact on vitamin B-6 status shown here, there are adverse consequences of this polymorphism when combined with riboflavin deficiency (40). We recently reported that, among those with deficient riboflavin status, the variant *MTHFR* 677TT genotype predisposes adults to a 3-fold higher risk of hypertension, whereas better riboflavin status was associated with a reduced genetic risk (41). Moreover, we previously showed that the blood pressure phenotype associated with this polymorphism is responsive to intervention with riboflavin (24, 30, 42).

The high prevalence of riboflavin deficiency in the present study, as in the NDNS, is of concern. Future dietary recommendations should consider that vitamin B-6 status is dependent on not only dietary vitamin B-6 but also adequate riboflavin intakes. Of note, correlations of dietary vitamin -B6 and riboflavin intakes with their respective biomarkers, PLP and EGRac, were strengthened when supplement users were included, perhaps pointing to the contribution of B-vitamin supplements in helping to maintain adequate nutrient status, albeit the wider range of biomarker concentrations could also have contributed to the stronger correlation observed. Furthermore, the regression analysis showed that age was an important determinant of PLP, but this relationship disappeared when supplement users were included, suggesting that older people in particular may benefit from B-vitamin supplementation to offset the age-related decline in PLP, as previously suggested (32, 43). Fortification could also have beneficial effects in optimizing the status of these B-vitamins, with previous evidence that PLP concentrations are higher, and EGRac lower, in regular consumers of fortified food (44). Unlike in the United Kingdom and Ireland, a mandatory enrichment program is in place in the United States and Canada, whereby riboflavin lost from grain during processing is added. Despite such policy, there is some evidence from a small cohort of Canadian females to indicate that low riboflavin is prevalent, suggesting that, at current levels, added riboflavin in North America may be insufficient to maintain adequate riboflavin status (45). Beyond the metabolic consequences (for PLP) of riboflavin deficiency, as shown here and previously (20, 21), there may be long-term adverse health impacts. We previously showed that low PLP concentrations at baseline predicted a greater rate of cognitive decline over a 4-y follow-up period among adults aged >60 y (14) and were associated with a 45% and 73% increased risk of depression and anxiety, respectively, in the TUDA study (15). Moreover, suboptimal riboflavin status is associated with a higher risk of anemia in females (29) and higher blood pressure in adults from 18 y when combined with the variant *MTHFR* 677TT genotype (41). Given the potential adverse health impacts, even in the absence of clinical deficiency, the high prevalence of riboflavin deficiency shown here and previously reported (36) requires further investigation.

The current study has a number of strengths. Although previous studies in this area were limited by small sample sizes (21, 31), we report the association of PLP with riboflavin in a large, well-characterized sample of >5000 adults (aged 18–102 y), including nationally representative data. Our study adds considerably to previous findings in relation to PLP in older adults (46) as regards its interrelationship with riboflavin. A particular strength was the use of EGRac, widely accepted as the gold-standard method for assessing riboflavin status (2). In addition, all analyses from the 3 component cohorts of the study were centralized to 1 laboratory using standardized protocols to ensure consistency. Moreover, corresponding dietary intake data were available for almost 1000 participants from a nationally representative cohort of Irish adults, enabling the effect of riboflavin and other determinants of PLP to be examined following adjustment for dietary vitamin B-6 intake. This is also the first study to consider the relationship of *MTHFR* genotype with these interrelated nutrients. The main limitation of this study is the observational design that does not allow conclusions to be drawn regarding the causality of the observed associations.

In conclusion, the current findings show that, independent of dietary vitamin B-6 intake, plasma PLP is strongly associated with riboflavin status across adulthood, confirming the known metabolic dependency of vitamin B-6 on FMN. Furthermore, the adverse impact of riboflavin deficiency on PLP appears to be exacerbated if combined with the *MTHFR* 677TT genotype. Our observations thus suggest that riboflavin is the limiting nutrient for maintaining adequate concentrations of PLP, particularly in older adults and in those with the variant *MTHFR* genotype. These findings have important implications for emerging dietary recommendations in age- and sex-specific subgroups in that they indicate that dietary riboflavin intake should be considered when setting vitamin B-6 recommendations. Randomized trials are, however, necessary to investigate the PLP response to riboflavin intervention at doses within the dietary range.

## Supplementary Material

nqac240_Supplemental_File

## Data Availability

Data described in the article, code book, and analytic code will be made available upon request, pending application and approval from the Irish Universities Nutrition Alliance (IUNA) Data Access Committee.

## Notes

This study was completed as part of the DERiVE project, awarded under the Joint Programming Initiative a Healthy Diet for a Healthy Life (JPI-HDHL) scheme for transnational research under the “Biomarkers for Nutrition and Health” scheme: UK–Biotechnology and Biological Sciences Research Council (BBSRC, grant ref: BB/P028241/1). The Trinity-Ulster Department of Agriculture (TUDA) and National Adult and Nutrition Survey (NANS) data were collected as part of an All-Ireland initiative under the Joint Irish Nutrigenomics Organisation (JINGO), supported by the Irish Department of Agriculture and Food, and the Marine and Health Research Board [under the Food Institutional Research Measure (FIRM)] and the Northern Ireland Department for Employment and Learning (under its Strengthening the All-Island Research Base initiative). The funders of this research had no role in the design, methods, subject recruitment, data collections, analysis, and preparation of paper. The Northern Ireland Department for the Economy (DfE) provided a PhD studentship for HJ.

Author disclosures: The authors report no conflicts of interest.

Supplemental Figure 1 is available from the “Supplementary data” link in the online posting of the article at https://academic.oup.com/ajcn/.

Abbreviations used: CRP, C-reactive protein; EGRac, erythrocyte glutathione reductase activation coefficient; MTHFR, methylenetetrahydrofolate reductase; NANS, National Adult and Nutrition Survey; NDNS, National Diet and Nutrition Survey; PLP, pyridoxal 5′-phosphate; PPO, pyridoxine 5′-phosphate oxidase; TUDA, Trinity-Ulster Department of Agriculture.
