# Supplementary material for: Vitamin B-6 and riboflavin, their metabolic interaction, and relationship with MTHFR genotype in adults aged 18–102 years
Source: Am J Clin Nutr. 2022 Oct 20;116(6):1767–78. doi: 10.1093/ajcn/nqac240 (PMC9761749; doi:10.1093/ajcn/nqac240)
Supplement: nqac240_Supplemental_File [file nqac240_supplemental_file.pdf]

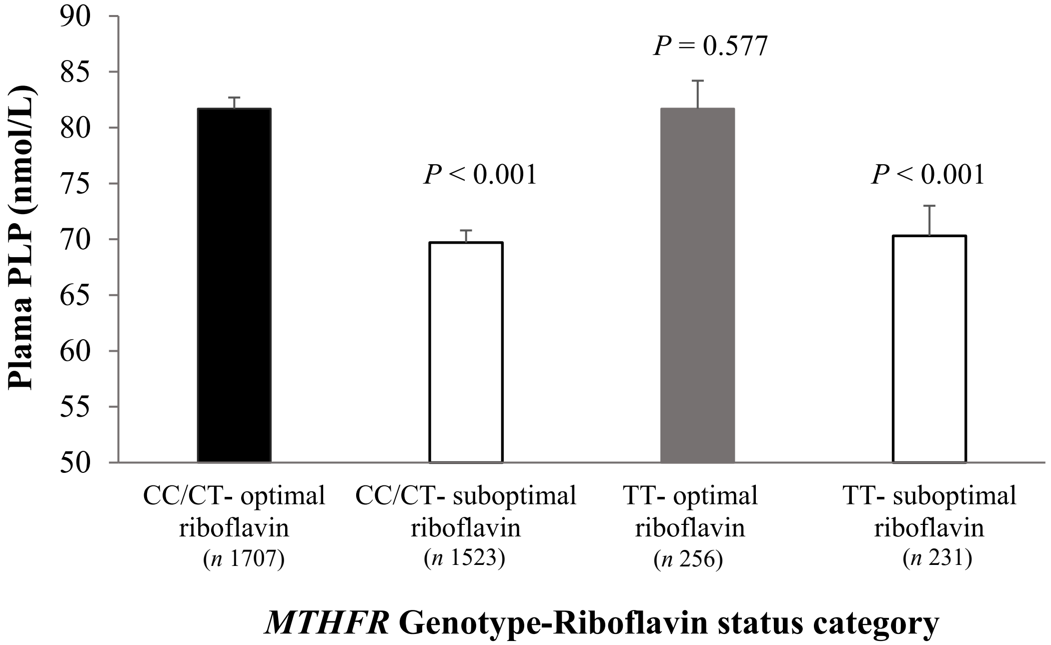

Supplemental FIGURE 1. Plasma PLP concentrations stratified by *MTHFR* genotype in optimal v suboptimal riboflavin status.

Riboflavin status defined as optimal (EGRac ≤ 1.26) and suboptimal (EGRac 1.27-1.39). *P* values refer to comparisons of PLP concentrations of each *MTHFR* genotype-riboflavin category relative to CC/CT riboflavin optimal status category (reference category), analyzed by ANCOVA controlling for age. Comparison of PLP concentrations between TT riboflavin-optimal versus TT riboflavin suboptimal: p=0.017. For this analysis, CC and CT genotype groups were combined as they are phenotypically similar, as we previously reported (Ward et al, 2020).

Abbreviations: *MTHFR*. *Methylenetetrahydrofolate reductase*; PLP, pyridoxal-5-phosphate
